# The upper-airway microbiota and loss of asthma control among asthmatic children

**DOI:** 10.1038/s41467-019-13698-x

**Published:** 2019-12-16

**Authors:** Yanjiao Zhou, Daniel Jackson, Leonard B. Bacharier, David Mauger, Homer Boushey, Mario Castro, Juliana Durack, Yvonne Huang, Robert F. Lemanske, Gregory A. Storch, George M. Weinstock, Kristine Wylie, Ronina Covar, Anne M. Fitzpatrick, Wanda Phipatanakul, Rachel G. Robison, Avraham Beigelman

**Affiliations:** 1 Department of Medicine, University of Connecticut, Farmington, CT USA; 2 The Jackson Laboratory for Genomic Medicine, Farmington, CT 06032 USA; 3 Department of Pediatrics, University of Wisconsin School of Medicine and Public Health, Madison, WI 53726 USA; 4 Department of Pediatrics, St. Louis Children’s Hospital, Washington University School of Medicine, St Louis, MO 63110 USA; 5 Department of Public Health Sciences, Penn State University, Hershey, PA USA; 6 Department of Medicine, University of California, San Francisco, CA 94143 USA; 7 Department of Medicine, St. Louis Children’s Hospital, Washington University School of Medicine, St Louis, MO 63110 USA; 8 Department of Medicine, University of Michigan, Ann Arbor, MI 48109 USA; 9 National Jewish Health, Denver, CO 80206 USA; 10 Department of Pediatrics, Emory University, Atlanta, GA 30322 USA; 11 Asthma, Allergy and Immunology, Boston Children’s Hospital, Harvard Medical School, Boston, MA 02115 USA; 12 Ann and Robert H Lurie Children’s Hospital of Chicago, Chicago, IL 60611 USA; 13 Kipper Institute of Allergy and Immunology, Schneider Children’s Medical Center, Tel Aviv University, 5891000 Tel Aviv, Israel

**Keywords:** Paediatric research, Translational research

## Abstract

The airway microbiome has an important role in asthma pathophysiology. However, little is known on the relationships between the airway microbiome of asthmatic children, loss of asthma control, and severe exacerbations. Here we report that the microbiota’s dynamic patterns and compositions are related to asthma exacerbations. We collected nasal blow samples (n = 319) longitudinally during a clinical trial at 2 time-points within one year: randomization when asthma is under control, and at time of early loss of asthma control (yellow zone (YZ)). We report that participants whose microbiota was dominated by the commensal *Corynebacterium* *+* *Dolosigranulum* cluster at RD experience the lowest rates of YZs (p = 0.005) and have longer time to develop at least 2 episodes of YZ (p = 0.03). The airway microbiota have changed from randomization to YZ. A switch from the *Corynebacterium* *+* *Dolosigranulum* cluster at randomization to the *Moraxella-* cluster at YZ poses the highest risk of severe asthma exacerbation (p = 0.04). *Corynebacterium’s* relative abundance at YZ is inversely associated with severe exacerbation (p = 0.002).

## Abstract (web summary)

How the airway microbiome influences asthma pathophysiology remains unclear. Here, the authors analyse nasal samples of cohort of school-age children with persistent asthma and find that the microbiota’s patterns and composition at time of early loss of asthma control associate with severe asthma exacerbations.

## Introduction

Asthma exacerbations have high impact on children, their families, the health care system, and may lead to subsequent decline in lung function^1,2^. Early signs of loss of asthma control, often referred to as the Yellow Zone (YZ), is a period during which the patient is at risk of symptom progression to severe exacerbation.

Among preschool children, the airway microbiome is associated with respiratory illness severity, future wheezing, and childhood asthma^3–7^. However, it is unknown if the airway microbiome is related to asthma control and risk of exacerbations among school-age children with mild asthma.

To investigate this question, we utilize a well-characterized cohort of school-age children with mild-moderate persistent asthma treated with daily ICS in a clinical trial^8^. We prospectively investigate whether the upper-airway microbiota at the time of respiratory health (randomization) is related to the development of future YZ episodes, and whether the microbiome at YZ are related to the likelihood of progression to severe asthma exacerbation requiring oral corticosteroids (OCS).

We report that the airway microbiota colonization patterns are differentially associated with risk of loss of asthma control and severe exacerbations. Specifically, airway microbiota dominated by *Corynebacterium* *+* *Dolosigranulum* genera are associated with favorable clinical outcomes compared to microbiota dominated by more pathogenic bacteria: *Staphylococcus, Streptococcus, and Moraxella*.

## Results

### Characterization of the airway microbiota at randomization

To first identify any cohort characteristics that affects airway microbiota, we performed Permutational multivariate ANOVA (PERMANOVA) using nasal blow samples collected at time of randomization (RD) from 214 children participating in a clinical trial (mean age 8.0 +/− 1.8 years, 59% were males, and 57% were Caucasian). The 214 children are a well representation of the total clinical trial cohort (*n* = 254) as demographics and clinical characteristics were not statistically different between the 254 children participated in the clinical trial and this subset of 214 children.

**Fig. 1:**
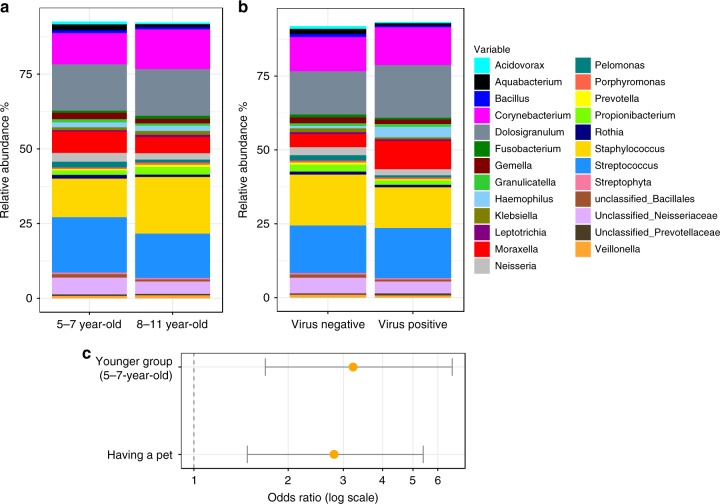
Top 25 major taxa identified at randomization. Top 25 most abundant taxa in different age groups (**a**) and by the presence of respiratory virus in the sample (**b**) are shown by barplots. The Odd Ratio of developing YZ is displayed with 95% confidence interval (**c**). Source data are provided as a Source Data file.

The overall microbial composition and abundances were significantly different in age groups (*p* = 0.02, PERMANOVA). Differential abundance analysis by DESeq showed that older children (8–11-year-old) had higher randomization abundance of *Staphylococcus* (*p* = 0.04), while younger children (5–7-year-old) had higher abundances of *Moraxella* (*p* = 0.05) and *Streptococcus* (*p* = 0.03) (Fig. 1a). Younger participants (5–7-year-old) were more likely to develop YZ (OR = 3.2, 95% CI 1.7–6.8, *p* = 0.003) compared to older participants. The presence of a pet in the house was associated with an increased risk of developing YZ (OR = 2.8, 95% CI 1.5–5.4, *p* = 0.006; (Fig. 1c)). 22.9% of the participants were treated with antibiotics during the 6 months prior to randomization, but this covariate did not affect the composition of the microbiota at time of randomization (*p* = 0.52, PERMANOVA). Other than age or the presence of pets, no other clinical variables (gender, ethnicity, BMI, total IgE level, number of asthma-related hospitalizations in the past year, peripheral eosinophil count, and lung function values (FEV1 percent, FEV1/FVC ratio) were found to be associated with risk of developing YZ. Based on these findings, all subsequent analyses that included randomization samples were adjusted for age and the presence of pets at home.

As it is well known that viral respiratory infections contribute to asthma exacerbations^9^, we investigated the effect of respiratory viruses in our cohort. Respiratory viruses were detected in 33% of the randomization samples: 64% of these viruses were rhinovirus. The proportion of virus positive samples was numerically higher in younger age group (39.3% in young vs. 28.0% in old groups), but was not significantly different (*p* = 0.11, Chi-squared test). Comparing to viral negative samples, the relative abundance of *Moraxella* (*p* = 0.04, by DESeq) were higher while the relative abundances of *Bacillus* (*p* = 0.03, by DESeq) and *Staphylococcus* (*p* = 0.04, by DESeq) were significantly lower in the viral positive samples, suggesting a possible interaction between viruses and these bacterial genera (Fig. 1b). However, the presence of respiratory virus at randomization (while the children did not have respiratory symptoms) was not associated with the risk of future YZ development (OR = 1.4, 95% CI 0.72–2.90, *p* = 0.33), or the risk of future exacerbations (OR = 1, 95% CI 0.39–2.44, *p* = 0.9). The finding that respiratory viruses were not predictive at baseline for subsequent loss of control and exacerbations may in part be attributable to the lack of differentiation of rhinovirus types as in children Rhinovirus C has been shown to be most strongly associated with asthma exacerbations compared to Rhinovirus A or B^10,11^. Nevertheless, all subsequent analyses that included randomization samples were adjusted for the presence of respiratory viruses at randomization.

### Baseline airway microbiota and future loss of asthma control

**Fig. 2:**
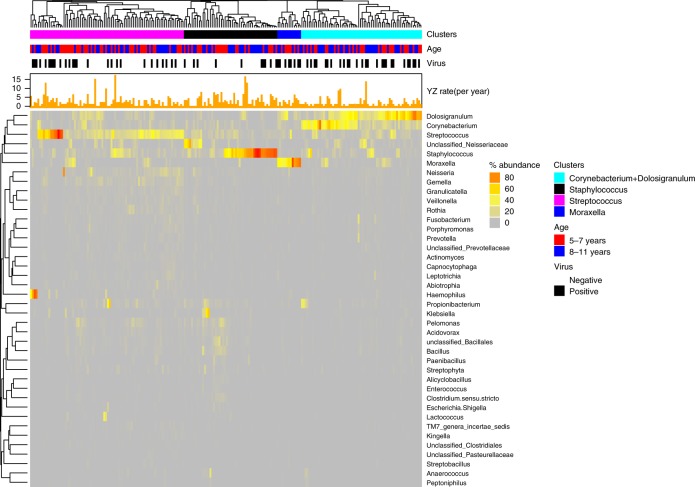
Bacterial clusters identified in nasal samples at randomization (*n* = 214). *Corynebacterium* + *Dolosigranulum* (cyan), *Staphylococcus* (black), *Streptococcus* (pink), and *Moraxella* (blue) were identified by hierarchical clustering and complete linkage approach. Bacterial genera with relative abundance >0. 1% was used for clustering analysis. Age group, presence of respiratory virus in the sample, and the annualized yearly rate of yellow zone episodes are annotated along each cluster. Source data are provided as a Source Data file.

**Fig. 3:**
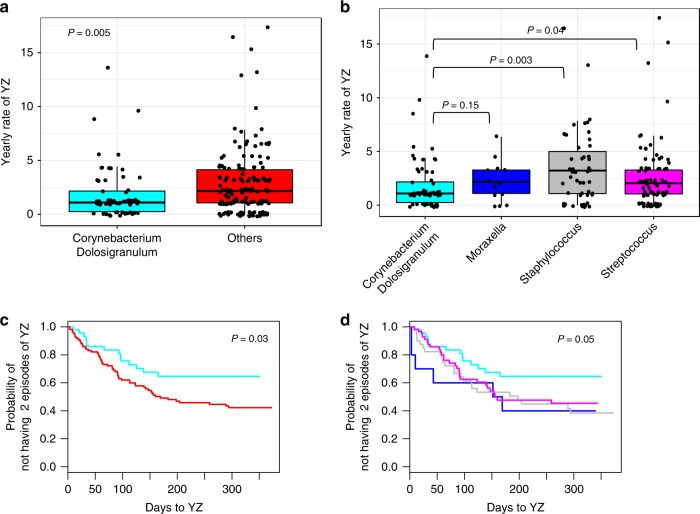
Yearly rate of yellow zone (YZ) in respiratory clusters at randomization. **a** Yearly rate of YZ in *Corynebacterium*+*Dolosigranulum* cluster compared to the combined *Others* 3 clusters. The line in the middle of a boxplot represents the median value of Yearly rate of YZ; the line at bottom and top of a box represent 25 and 75 percentile of the data value. Dots above the vertical line represent outliers of the data. **b** Yearly rate of YZ in the four respiratory clusters. In **a**, **b**, each dot represents a participant in the cluster. Statistical significance of YZ rate differences between clusters was tested using Wilcoxon rank-sum test. **c** *Corynebacterium* + *Dolosigranulum* cluster has significantly lower probability than the combined other three clusters (Other) to develop >=2 episode of YZ (*p* = 0.02 by Cox Proportional-Hazards model). **d** *Corynebacterium* + *Dolosigranulum* cluster has significantly lower probability than the *Moraxella* cluster and the *Staphylococcus* cluster to develop >=2 episodes of YZ (*p* = 0.05 by Cox Proportional-Hazards model). The colors of the clusters in **c**, **d**, are as in **a**, **b**. Source data are provided as a Source Data file.

To first evaluate whether the microbiota at time of well-controlled asthma (randomization) is related to loss of asthma control at YZ, we perform unsupervised hierarchical clustering analysis using 214 nasal samples from randomization. We identified four clusters dominated by the following genera: *Corynebacterium* + *Dolosigranulum*, *Staphylococcus*, *Streptococcus*, and *Moraxella* (Fig. 2). During the follow up period (320 days), 75.7% and 43.4% of the participants experienced at least one and two episodes of YZ, respectively. The median annualized rate of YZ was significantly lower in participants who had the *Corynebacterium* + *Dolosigranulum* dominated cluster at randomization compared to the aggregate of the 3 other clusters combined together, or any other cluster (Fig. 3a, b; *p* = 0.005; Wilcoxon rank-sum test). In addition, Cox Proportional-Hazards analysis showed that participants in the *Corynebacterium* *+* *Dolosigranulum* cluster had significantly longer time to develop at least 2 episodes of YZ compared to participants in the combined 3 other clusters (Fig. 3c, *p* = 0.03, ward test) and compared to any other cluster (Fig. 3d, *p* = 0.05, ward test). Time to the first episode of YZ, was not statistically different between the groups.

### Changes in airway microbiota from randomization to YZ

**Fig. 4:**
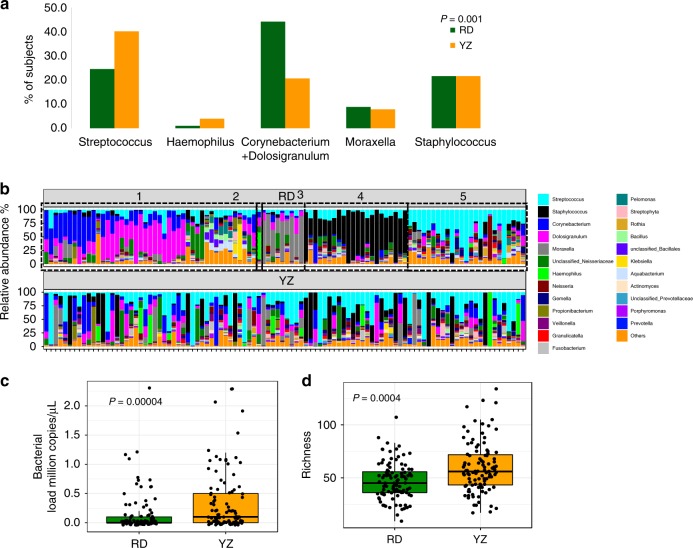
Bacterial microbiome comparison between randomization (RD) and yellow zone (YZ). **a** Five bacterial clusters were identified using the paired randomization (*n* = 102) and YZ data (*n* = 102). Each barplot represents the proportion of patients belonging to a given cluster. Green-samples collected at RD, and orange-samples collected at YZ. Sums of proportions for green/orange bars is equal to 100%. The statistical difference of patient distribution across the five clusters was identified using Chi-Square test. **b** Changes in the relative abundance of bacterial microbiome from RD to YZ in each of the study participants. The relative abundance of the top 25 bacteria from RD (top panel) and YZ (bottom panel) in the same subjects are plotted. The samples are ordered by clusters at RD from left to right (1) *Corynebacterium* + *Dolosigranulum* (2) *Haemophilus* (3) *Moraxella* (4) *Staphylococcus* (5) *Streptococcus clusters*. **c** Total bacterial load in RD and YZ samples. Total bacterial load is represented as 16S rRNA million copies per μL, estimated by qPCR. **d** Bacterial richness at RD and YZ. Statistical difference of bacterial load or richness was tested using Wilcoxon rank-sum test. Source data are provided as a Source Data file.

**Fig. 5:**
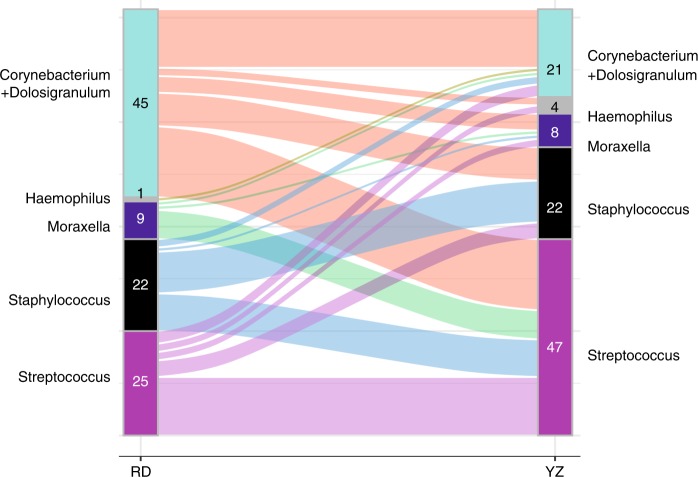
Changes in bacterial clusters from randomization to yellow zone YZ. The numbers within each cluster at RD (left) and YZ (right) are the number of patients within each specific cluster switch. Source data are provided as a Source Data file.

We next determined the dynamics of nasal bacterial microbiota from randomization to YZ utilizing samples from the 102 participants who contributed samples both at randomizing and at YZ (102 paired samples (total 204 samples)). The bacterial compositions and their relative abundance in individual patients demonstrated profound alteration from randomization to YZ (Figs. 4b, 5). Strikingly, more than half of the patients switched to a different dominant cluster between these 2 time points, most commonly to the *Streptococcus* cluster (Fig. 5). Resultantly, *Streptococcus* cluster became the most prevalent cluster at YZ (Fig. 4a). The changes of microbiota are also evident in bacterial alpha diversity and total bacterial load. At YZ, total bacterial load (Fig. 4c) and bacterial richness (Fig. 4d) were significantly higher (*p* < 0.01 for both, Wilcoxon rank-sum test) than those at randomization.

### Airway microbiota at YZ and asthma exacerbations

**Fig. 6:**
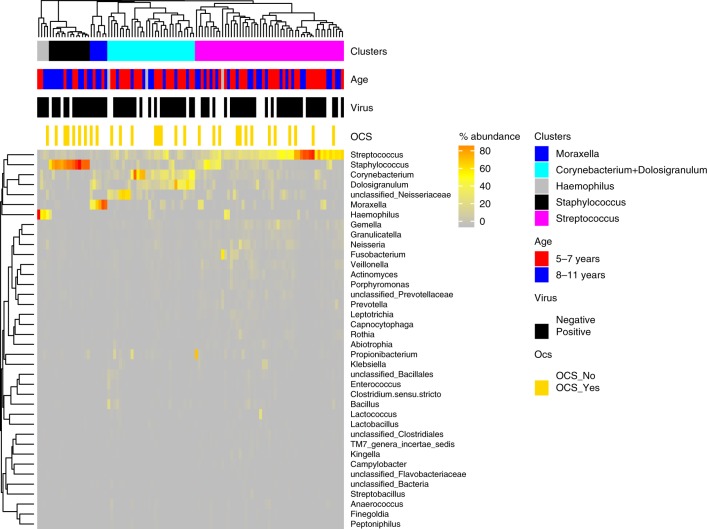
Bacterial clusters identified from 105 nasal samples at yellow zone (YZ). Five clusters, *Corynebacterium* + *Dolosigranulum* (cyan), *Staphylococcus* (black), *Streptococcus* (pink), *Moraxella* (blue), and *Haemophilus* (gray) were identified by hierarchical clustering and complete linkage approach. Bacterial genera with relative abundance >0.1% was used for clustering analysis. Source data are provided as a Source Data file.

**Fig. 7:**
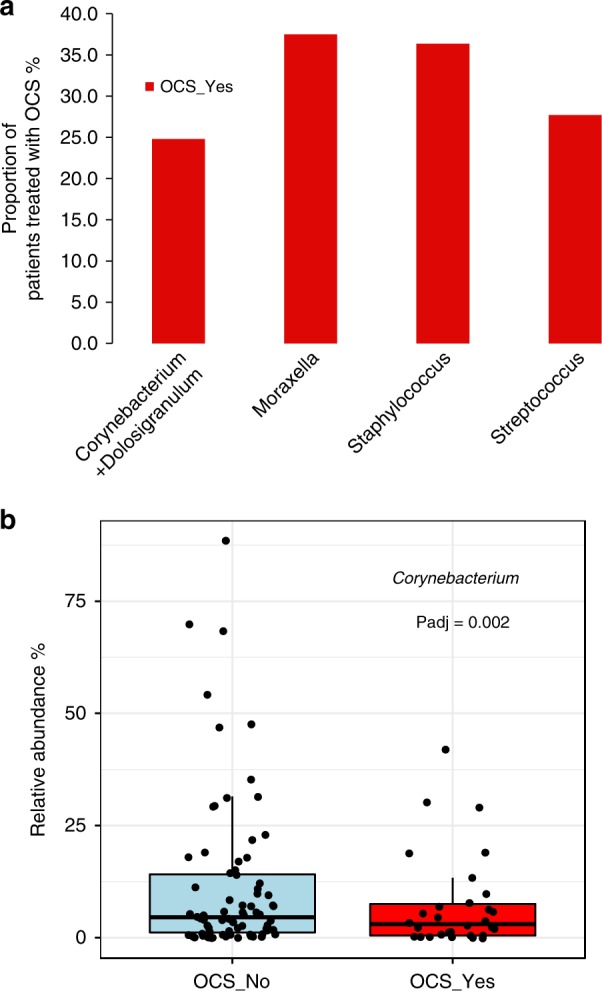
The microbiome at YZ and the outcome of severe exacerbations (OCS treatment). **a** The proportion of patients progressed to severe exacerbation (OCS therapy, *n* = 30) across different bacterial clusters at YZ (**b**). The relative abundance of *Corynebacterium* at the time of YZ is significantly lower in patients that progressed to severe exacerbation (*P* = 0.002, Deseq). Source data are provided as a Source Data file.

To test whether the microbiome at the time of YZ is associated with severe exacerbation, we compared the microbiota difference in participants who progressed to severe exacerbation requiring OCS therapy (30/105 = 28.6%) and those who did not (75/105 = 71.4%) (Fig. 6). We found a numerically lower, but not statistically significant, proportion of patients who required OCS from the *Corynebacterium* *+* *Dolosigranulum* cluster at the time of YZ compared to the other three clusters (Fig. 7a, *p* = 0.35, Chiseq-test). Furthermore, analysis of specific bacterial genera revealed that *Corynebacterium* was more abundant at YZ in samples obtained from episodes that did not progress to severe exacerbation (Fig. 7b, *P* = 0.002, DeSeq). In addition, higher relative abundance of *Corynebacterium* was associated with a modest reduction in the risk of progressing to exacerbation (OR = 0.92, 95%CI 0.89–0.94, *P* = 0.04). The reason that this effect was not very strong, may be related to the relatively low rate of participants who progressed to severe exacerbation, leading to reduced power for the YZ analyses. We did not find any association between that microbiome at randomization and severe exacerbations, likely due to the profound changes in the microbiome between RD and YZ. Finally, bacterial richness (*p* = 0.64, Wilcoxon rank-sum test) or load (*p* = 0.16, Wilcoxon rank-sum test) at yellow zone were not associated with asthma exacerbations.

### Dynamic change of the microbiota and asthma exacerbations

Given the upper airway microbiota of most participants changed from randomization to YZ(Fig. 5), we next investigated whether the dynamic change of the microbiota, namely, switching to a different cluster or maintaining of the same cluster was associated with exacerbation risk. Switching from *Corynebacterium* + *Dolosigranulum* cluster at randomization to the *Moraxella* cluster at YZ was associated with the highest risk of exacerbation compared to the other combinations of cluster changes (*P* = 0.04, Chiseq-test).

### Respiratory viruses at YZ and asthma exacerbations

**Fig. 8:**
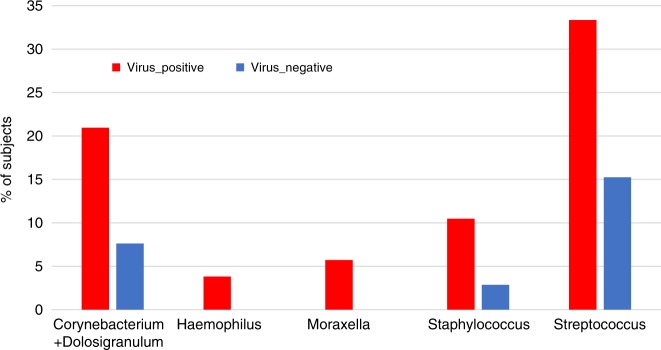
Proportion of subjects positive or negative for respiratory virus across the five clusters at YZ. The number of virus positive ((red, *n* = 78) and virus negative (blue, *n* = 27) samples are distributed disproportionally across different clusters. Subjects belonging to *Haemophilus* and *Moraxella* clusters were all virus positive. Source data are provided as a Source Data file.

We next evaluated the potential contribution of respiratory viruses at time of YZ to asthma exacerbations. Respiratory viruses were detected in 78 (74.3%) of the YZ samples, of these 48 samples were enterovirus/human rhinovirus (EV/RV) positive. Presence of virus was not associated with severe exacerbations, likely due to the very high viral detection rate in these samples and a very few samples without viruses at the time of YZ, all of which resulting in a reduced power for this analysis. Samples that belonged to *Moraxella* (*n* = 4) and *Haemophilus* clusters (*n* = 6) were all virus positive (Fig. 8). Interestingly, most of the samples assigned into *Moraxella* and *Haemophilus* dominated microbial clusters at YZ switched from a non-*Moraxella* and non-*Haemophilus* clusters at RD (Fig. 5), reflecting the emergence of *Moraxella* and *Haemophilus* communities at the time of YZ.

## Discussion

In this prospective, longitudinal study we characterized the upper-airway bacterial microbiota of school-age children receiving daily low-dose ICS at time of well-controlled asthma (randomization) and during loss of asthma control (YZ). We found that the airway microbiota colonization patterns were differentially associated with risk of loss of asthma control and severe exacerbation. Specifically, the airway microbiota of children classified as dominated by *Corynebacterium* *+* *Dolosigranulum* genera during randomization was associated with a lower risk of developing loss of asthma control compared to those with microbiota being dominated by more pathogenic bacteria, specifically *Staphylococcus, Streptococcus, and Moraxella*. Furthermore, at the time of YZ, *Corynebacterium’s* relative abundance was inversely associated with the likelihood of progressing from YZ to severe exacerbation.

Previous studies found that colonization of the upper airways with opportunistic bacterial pathogens including *Streptococcus, Moraxella, and Haemophilus* is more common among asthmatics than healthy individuals^12–14^. Our findings, that still needs to be validated in independent cohorts, suggest the potential pathogenic role of these bacteria in asthma. These finding is in one line with a recent study^15^, in children with moderate-severe asthma, which revealed that a *Moraxella* dominated upper airway microbiome was associated with increased exacerbation risk. Our findings extend the potential pathogenic role of *Moraxella* to the large population of children with mild asthma, which are the vast majority of childhood asthma patients. Further, our study revealed that *Staphylococcus* was not protective, but rather, was associated with highest YZ episodes, which is different from previous studies^3,6,15^. This difference may be related to different age groups, different disease severity, or different *Staphylococcus* species^3,16^. While uncharacterized previously, the vast majority of *Staphylococcus* genera in our study were comprised of *Staphylococcus aureus*, which was previously reported to be associated with wheeze and asthma among children and young adults^17^.

Our findings highlight the association between commensal bacteria such as *Corynebacterium* and *Dolosigranulum* and asthma control*. Corynebacterium* is the most abundant genus identified in the nose by the Human Microbiome Project that characterized the normal microbial composition in healthy adults^18^, and was found less frequently as a dominant member of the nasal microbiome in asthmatic adults^13^ suggesting that this bacteria may have a protective effect. Since bacteria colonizing the upper airways, as those colonizing other niches within the human body, exist in a competitive state and it is plausible that competitive colonization may be one of the factors by which commensal bacterial provide protection against pathogen colonization and overgrowth^19^. Indeed, *Corynebacterium* and *Dolosigranulum* can inhibit the growth of *Streptococcus* by releasing antibacterial products that may prevent nasal colonization with *Streptococcus*^20,21^. Collectively these observations indicate that from an ecological perspective, a microbiome at equilibrium may resist colonization with pathogenic bacteria and is important for the maintenance of a healthy airway. This hypothesis is supported by the finding of our study as the composition of airway microbiota at time of respiratory health was associated with loss of asthma control during the following year. Validation this finding by an independent cohort may lead to identification of potential microbiome markers at respiratory health to predict loss of asthma control in future.

The finding of the current study in school-aged children expand the findings of previous work by Teo et al.^3,6^ who showed that among young preschool children, detection of *Streptococcus, Moraxella*, and *Haemophilus*, as a dominant members of the upper airway microbiota, was more common during viral upper-respiratory infections compared to times of respiratory health, and predicted progression to lower respiratory symptoms. In addition, microbiota characterized by dominant *Corynebacterium, Staphylococcus*, and *Alloiococcus* (*Dolosigranulum* in some databases) were more common during well-visits supporting the potentially protective role of *Corynebacterium*, and *Dolosigranulum* identified in our study.

The airway microbiota composition dramatically changed between randomization and YZ visits. The directionality of switch was found to be important, since switching from the microbiota dominated by *Corynebacterium*/*Dolosigranulum* to *Moraxella* cluster was associated with higher risk of asthma exacerbation. This observation indicates that commensal nasal microbiota in asthmatic children does not appear to prevent overgrowth by all pathogenic bacteria, in particular *Moraxella*, suggesting distinct intricate microbial interactions between specific members of the airway microbiota, which remain to be elucidated. In addition, respiratory viruses, mainly rhinovirus, were detected in most YZ samples. Although limited by small sample size, an interesting observation is that samples belonging to the *Moraxella* or *Haemophilus* dominated microbiota during YZ were all positive for respiratory viruses. This finding is consistent with the high rate of virus-positive samples in children with these microbiota signatures at randomization, and with previous reports linking rhinovirus infection with the detection of these bacterial genera in the airway^22^.

This study highlights some bacteria that were associated with favorable asthma outcomes, while other bacteria were associated with asthma morbidity. However, the design of this current study does not allow to determine causality. It is still unknown if the microbiome changes drive asthma activity, are consequence or the trigger of viral infection, or are result of bidirectional cross-talk between the microbiome and host mucosal and systemic immune response during YZ development and acute exacerbations. It may also be that the microbiota changes reflect lack of asthma control and airway inflammation. More frequent sampling, together with simultaneous host immune studies, may allow insight into these interactions. In addition, studies in mice infected with these bacteria may allow to determine causality.

The advantages of this study are mainly related to its conduct as a study coupled to a well-designed clinical trial^8^. Patients were carefully characterized resulting in a homogenous population of school-aged children all requiring step 2 asthma care, treated with an identical dose of ICS. Therefore, we minimized microbiota differences that may be related to disease severity and/or effects of different ICS dosing, which are factors known to affect the airway microbiome^23^. Prospective data and sample collection as part of a clinical trial together with tight follow-up visits and calls have minimized recall and measurement biases. Finally, YZ samples were collected before applying study intervention (high-dose ICS) eliminating the effect of high-dose ICS on the microbiome.

This study has some limitations. The parent STICS clinical trial^8^ had a relatively low rate of YZ episodes, and as expected only 30% of YZ episodes progressed to severe exacerbation, leading to reduced power for the YZ analyses. In addition, a follow-up nasal sample after the YZ was not obtained, and this could have provided important information related to the microbiota composition at the time of resolution of the episode. One additional factor that may be related to the microbiome composition is allergen exposure in atopic children. As we do not have allergen exposure data, we cannot comment on this relationship in our study.

In this study, we investigated associations between bacterial clusters, defined by the relative abundance of the bacteria, and clinical outcomes. Determining the absolute density of different bacteria in a metagenomic sample is an emerging concept in microbiome research, which has showed interesting results in a stool microbiome study^24^. We view the relative and absolute abundances of the microbiome as complementary to each other. As a low biomass material, bacterial density from nasal wash warrants thorough investigation in future regarding methodology including efficiency and robustness and data normalization for microbiome analysis. In addition, functional level characterization of the bacterial microbiome by transcriptome analysis or IgA-Seq are likely to improve our understanding of the role of airway microbiome in asthma and its exacerbation.

This study revealed that changes in the upper respiratory tract are associated with events in the lower respiratory tract. Although there are similarities between the lung and upper-airway microbiota compositions, studies have shown that these different compartments have different microbiome composition^13,25^ Therefore, we must acknowledge that changes in the nasal microbiota may represent a secondary phenomenon and are not necessarily related to asthma control. However, some key bacterial taxa co-exist in the nasal and bronchial airways, especially in asthmatics^13^. In addition, multiple studies have highlighted the relevance of the upper airway microbiota as a surrogate for the lung microbiota, and have shown that the upper-airway is a relevant compartment that provides valuable data on asthma inception^3,26^, asthma diagnosis^14,27^, and asthma exacerbations^22,27^.

In summary, we demonstrate a relationship between upper-airway microbiota composition and the risk of both loss of asthma control and severe exacerbations, among school-aged children with asthma. A randomization upper airway microbiota dominated by *Corynebacterium* *+* *Dolosigranulum* was associated with a significantly lower rate of YZ development. In addition, upper-airway microbiota composition was not static, and a shift to a *Moraxella*-dominant microbiome at YZ and/or a lower *Corynebacterium* abundance at YZ were both associated with increased risk of severe exacerbations during the following year. These findings still need to be validated in independent cohorts. Then, there is a need to perform animal studies to further investigate the role of the bacteria identified in this study in asthma exacerbations. If their role are confirmed, investigation of more advanced strategies that modulate microbiota composition would be indicated, in an effort to reduce the risk of asthma exacerbations.

## Methods

### Study design and participants

This microbiome study was a microbiome study coupled to the Step Up Yellow Zone Inhaled Corticosteroids to Prevent Exacerbations (STICS; NCT02066129) clinical trial, conducted by the NHLBI’s AsthmaNet^8^. The STICS clinical trial was approved by the AsthmaNet steering committee, protocol review committee, and data and safety monitoring board (DSMB). The IRB’s of all AsthmaNet sites reviewed and approved the study protocol. We have obtained informed consent from all participants.

The STICS clinical trial investigated whether, in school age children with mild-moderate persistent asthma who are treated with daily low-dose ICS, quintupling the dose of ICS in the YZ would reduce the rate of severe asthma exacerbations treated with oral corticosteroids.

A detailed description of the STICS clinical trial design, YZ criteria, study participants characteristics, and its results are detailed elsewhere^8^. Briefly, 254 children, 5–11 years of age, were treated for 48 weeks with maintenance open-label low-dose inhaled glucocorticoids (fluticasone propionate, 88 μg twice daily) and were randomly assigned to receive either the same ICS dose or use a quintupled dose ICS for 7 days at the early signs of loss of asthma control (YZ). The primary outcome was the rate of severe asthma exacerbations treated with systemic glucocorticoids, which were prescribed as a rescue therapy based on pre-specified protocol criteria. The rate of severe asthma exacerbations was not different between the groups^8^. This microbiome study, which was coupled to the STICS clinical trial, was conducted in compliance with all ethical regulations. The IRBs of all AsthmaNet clinical sites approved these microbiome investigations as part of the parent STICS trial, and informed consent was obtained from all study participants.

Nasal blow samples for microbiome studies were obtained^8,28^, at 2 time points: (1) At the randomization visit (RD) once the child had no respiratory symptoms. (2) At the time of the first episode of early signs of loss of asthma control (YZ) prior to starting the YZ intervention (regular or high-dose ICS). The second sample was obtained before starting the YZ intervention in order to avoid potential effect of high-dose ICS on the nasal microbiota. The YZ sample was obtained by the parents at home based on instructions received at the randomization visit. The nasal samples were analyzed for common respiratory viruses by multiplex polymerase chain reaction (respiratory MultiCode assay; EraGen Biosciences, Madison, WI) as previously reported^29^

### 16S rRNA gene sequencing and normalization

Total genomic DNA was extracted from 200 μl nasal blow samples using the bioMerieux NucliSENS easyMAG automated extractor kit following standard protocol. We followed standard Illumina sequencing protocol^30^. In brief, to characterize the bacterial microbiota, the V1 to V3 regions of 16S rRNA gene were amplified (primers 27F and 534R (27F:5′-AGAGTTTGATCCTGGCTCAG-3′ and 534R: 5′-ATTACCGCGGCTGCTGG-3′), barcoded, and sequenced on the Illumina Miseq (2 × 300 bp) platform. Paired-end reads were assembled using Flash V1.2.7. Assembled reads were assigned to taxonomies using Ribosomal Database Project (RDP) software with classification confidence at >=0.8. The processed reads were subsampled to 10,000 reads/sample for 16S rRNA gene sequences. We included extraction control, PCR negative control for DNA extraction and sequencing. Less than five hundred reads were found in these negative controls, suggesting background noise is less likely to have significant impact on the data analysis. To minimize the effect of background noise on data analysis, we additionally removed taxa that are potentially contamination from downstream analysis. These taxa include unclassified_*Bradyrhizobiaceae*, *Thermohydrogenium, Aquabacterium, unclassified_burkholderiales, Brevundimonas, Rhizobium*, and *Soonwooa*.

We used taxa with >=0.1% relative abundance (after removing the contamination taxa discussed above) to construct the heatmaps at randomization and yellow zone. These taxa accounts for 99% of total bacterial abundance on average (the minimal coverage is 82%).

To classify *Staphylococcus* to species level, we blasted all the reads that mapped to *Staphylococcus* genus to 16S rRNA database in NCBI. V13 reads with ~500 bp in length of *Staphyloccus* aligned to *S. aureus* with 100% coverage, 100% identity, which is distinct from *S. epidermidis* (97% identity).

### Quantification of bacterial load

Quantification of the bacterial 16S rRNA gene copy number in nasal blow samples was performed using a modification of the BactQuant assay^31^. Briefly, total nucleic acids were extracted from the samples using the automated BioMerieux NucliSens easyMAG extractor. The BactQuant quantitative PCR was performed on the extracted DNA as described by Liu et al.^31^ with the following modifications: total reaction volume was 20 μl, including 3 µl of extract, and the assay was performed on an Applied Biosystems 7500 Real Time PCR System instrument. Quantification standards consisted of dilutions of a plasmid containing the E. coli 16S rRNA gene with results being expressed as copies of 16S rRNA gene per microliter.

### Statistical analysis of the microbiome data

The processed reads were subsampled to 10,000 reads/sample for 16S rRNA gene sequences. The abundance of a taxon in a sample was represented as the relative abundance, which was calculated by dividing the number of reads assigned to a taxon by the total read counts, divided by 10,000, of the sample.

We performed both exploratory multivariable analysis and formal statistical testing. Exploratory multivariable analysis was done through hierarchical clustering to identify microbiome distribution patterns. Taxa with the relative abundances >0.1% was included in hierarchical clustering. Complete linkage was used for assigning samples to clusters^16^. R package ComplexHeatmap was used for cluster visualization and annotation of clinical variables. The names of clusters are defined based on the dominant bacteria genus in for that cluster. Permutational multivariate ANOVA (PERMANOVA) was used for formal statistical testing to investigate whether the bacterial community structure varied between different clinical parameters. DESeq2 was used to identify differential taxa between samples at randomization (RD) and YZ. Clinical variables including age, viral infection, gender and having a pet were first tested individually using PERMANOVA or DESeq2. The confounding variables were included along with variable of interest (RD and YZ) in the final model of PERMANOVA or DESeq2. The results from DESeq2 were further speculated by plotting the raw and relative abundance data. Results that are likely driven by outliers were removed from final reporting. Bacterial diversity including Richness and Shannon Diversity was computed using R package Vegan and statistical significance between groups was determined using Wilcoxon-rank test. Comparison of patient numbers between clusters was performed by chi-square or Fisher’s exact test. Exacerbation data was treated as categorical data (0 and 1), and was applied to a generalized logistic regression model with binomial distribution to determine whether a given taxon is associated with exacerbation outcome. Odd-Ratios (ORs) were evaluated using generalized logistic regression. Multivariate models of Cox Proportional-Hazards analysis was performed to assess the association between the microbiome clusters at randomization and the development of >=2 episodes of YZ after adjustment for age and the presence of pets. Kaplan-Meier survival analysis was also performed to view the results from Cox Proportional-Hazards analysis. A *P* value of less than .05 was considered statistically significant in all the analysis. *P* values were corrected by false discovery rate when multiple comparisons were involved. All the analyses, described above, were performed in R (version 3.2.2).

## Supplementary information

### Source data


Source Data


## Data Availability

Raw sequencing data of 16s rRNA gene are available from the SRA database with accession number PRJNA448764. The source data from Fig. 1–8 are provided as a Source Data file. All other data are available from the corresponding author upon reasonable requests.

## Code availability

Computational code from the study is available from the corresponding author upon request.
